# The TEAD Family and Its Oncogenic Role in Promoting Tumorigenesis

**DOI:** 10.3390/ijms17010138

**Published:** 2016-01-21

**Authors:** Yuhang Zhou, Tingting Huang, Alfred S. L. Cheng, Jun Yu, Wei Kang, Ka Fai To

**Affiliations:** 1 Department of Anatomical and Cellular Pathology, State Key Laboratory of Oncology in South China, Prince of Wales Hospital, The Chinese University of Hong Kong, Hong Kong, China; zyhjoe@gmail.com (Y.Z.); huangtingting0531@gmail.com (T.H.); 2 Institute of Digestive Disease, Partner State Key Laboratory of Digestive Disease, The Chinese University of Hong Kong, Hong Kong, China; alfredcheng@cuhk.edu.hk (A.S.L.C.); junyu@cuhk.edu.hk (J.Y.); 3 Li Ka Shing Institute of Health Science, Sir Y.K. Pao Cancer Center, The Chinese University of Hong Kong, Hong Kong, China; 4 Shenzhen Research Institute, The Chinese University of Hong Kong, Shenzhen 518000, China; 5 School of Biomedical Sciences, The Chinese University of Hong Kong, Hong Kong, China; 6 Department of Medicine and Therapeutics, The Chinese University of Hong Kong, Hong Kong, China

**Keywords:** TEAD proteins, transcription factor, Hippo pathway, YAP, TAZ, vgll

## Abstract

The TEAD family of transcription factors is necessary for developmental processes. The family members contain a TEA domain for the binding with DNA elements and a transactivation domain for the interaction with transcription coactivators. TEAD proteins are required for the participation of coactivators to transmit the signal of pathways for the downstream signaling processes. TEADs also play an important role in tumor initiation and facilitate cancer progression via activating a series of progression-inducing genes, such as *CTGF*, *Cyr61*, *Myc* and *Gli2*. Recent studies have highlighted that TEADs, together with their coactivators, promote or even act as the crucial parts in the development of various malignancies, such as liver, ovarian, breast and prostate cancers. Furthermore, TEADs are proposed to be useful prognostic biomarkers due to the ideal correlation between high expression and clinicopathological parameters in gastric, breast, ovarian and prostate cancers. In this review, we summarize the functional role of TEAD proteins in tumorigenesis and discuss the key role of TEAD transcription factors in the linking of signal cascade transductions. Improved knowledge of the TEAD proteins will be helpful for deep understanding of the molecular mechanisms of tumorigenesis and identifying ideal predictive or prognostic biomarkers, even providing clinical translation for anticancer therapy in human cancers.

## 1. Introduction

In mammals, there are four highly conserved TEAD/TEF transcription factors (*TEAD1*, *TEAD2*, *TEAD3*, *TEAD4*). These proteins share in common the TEA DNA binding domain, and are referred to as the TEA domain (TEAD) proteins, and require transcriptional coactivators for transcription activation [1]. Three broad groups of candidates for these coactivators have been identified and classified by Pobbati *et al.* including YAP1 (Yes-associated protein), TAZ (transcriptional coactivator with PDZ-binding motif), vgll proteins, and p160 family of nuclear receptor coactivators [2]. With the help of coactivators, the TEAD family plays a crucial role in some physiological processes as well as in human cancers. Therefore, a comprehensive and in-depth molecular understanding of TEAD proteins is required for further investigation and clinical translation. In this brief review, we summarize the roles of TEADs in malignancies and provide some clues on TEAD regulation in order to guide further studies. The increasing understanding of TEAD proteins will undoubtedly broaden our knowledge of their roles in tumorigenesis and help to identify new prognostic biomarkers or develop rational design of novel therapies.

## 2. The Structures of TEAD Proteins and TEAD-Coactivator Complexes

There are four genes in mammals, named *TEAD1*–*4*, that encode four homologous members in the TEAD family. TEAD family members share the same domain structure: a DNA-binding TEA/ATTS domain in the N-terminus that adopts a homeodomain fold [3], and an immunoglobulin-like β-sandwich fold in the C-terminus [4]. The N-terminus domain binds with DNA fragments like 5′-GGAATG-3′, which are present in the SV40 enhancer and the promoter regions of TEAD target genes [5]. The C-terminus functions as a transactivation domain for its recruitments of transcriptional coactivators [6]. TEAD proteins alone are incapable of inducing genes expression and need additional coactivators to achieve their transcriptional potential [1]. Coactivators can not directly bind to the DNA, but they can bind with transcription factors to activate the transcription process. Several coactivator candidates for TEADs have been identified including YAP and its paralog TAZ, vgll proteins, and the p160 family of nuclear receptor coactivators [2]. The three-dimensional structure of the YAP-TEAD1 complex in human and YAP-TEAD4 complex in mouse have been identified [7,8].

In the N-terminus, YAP and TAZ share a common TEAD-binding motif. The WW domains are located near the TEAD-binding motif. YAP has two isoforms, YAP1 and YAP2, according to the number of WW domains, but all TAZ orthologs have only one WW domain. In the C-terminus of YAP/TAZ, there is an activation domain [9]. TEAD contains an N-terminal TEA DNA-binding domain and a C-terminal region that contributes to YAP interaction [3,4]. In Li *et al.*’s study, they first uncovered the crystal structure encompassing YAP2 (residues 50–171) and TEAD1 (residues 194–411) complex. In the three-dimensional structure, YAP wrapped around the globular structure of TEAD1 thus forming extensive interactions through three interfaces with high conservation. The interface 3 in these interfaces was found to be the most important for the complex formation. In the same study, the functional residues of TEADs with YAP-binding interfaces were confirmed. Y406A and Y406H mutations strongly reduced the interaction with YAP, which also demonstrated the critical role of the interface 3 in the YAP-TEAD association (Y406 was in interface 3) [7]. Chen *et al.* demonstrated the crystal structure of the interaction between the YAP1 N-terminal domain and the TEAD4 C-terminal domain. They pointed out that the YAP N-terminus was folded into two short helices with an extended loop containing a PXXΦP motif. This motif is essential for the interaction of YAP with TEAD4. They also confirmed that the TEAD4 mutants, K297A, W299A, and Y429A, greatly abrogated the interaction of TEAD4 with YAP [8].

## 3. TEAD Transcription Factors in Physiological Process

TEAD transcription factors, which are also known as transcription enhancer factors (TEFs), were firstly identified by Xiao *et al.* in 1987. They were initially identified as nuclear proteins which could bind to the overlapping motifs in the domain B1 of the SV40 enhancer and acted as positive trans-acting enhancer factors [5]. Subsequently, TEAD1 was reported to bind human papillomavirus-16 (HPV-16) enhancer and activate the oncogenes E6 and E7 of HPV-16 [10]. TEADs are highly conserved transcription factors during evaluation and their homologs can be detected in almost any eukaryotes [11,12]. However, each TEAD member exhibits a distinct expression pattern, suggesting that each member has a unique function [12,13].

TEADs are required for cardiogenesis [14], myogenesis [15], development of neural crest [16], notochord [17] and trophoectoderm [18]. In development stages, TEAD proteins are significantly expressed in very early stages and their expression levels have already been detected in a two-cell stage embryo [13]. This result was further confirmed by other mice experiments in which no matter whether exogenous (injected plasmids) or endogenous, the mTEAD (mouse TEAD)-dependent enhancer, the activity of which depends on mTEAD DNA binding sites, could not be utilized until the two-cell stage of development [19,20]. Accordingly, mTEAD2 was the most significant member in the TEAD transcriptional factor family, for its activity was predominantly detected in the early mouse embryos. The expression of mTEAD1 or 3 were relatively low and mTEAD4 RNA was hardly detected in either oocytes or preimplantation embryos [13].

The physiological function of TEAD genes were deduced in mice through gene inactivation studies. Null mutation of TEAD1 resulted in embryonic lethal and heart developmental defect [14]. The findings also suggested that TEAD1 promoted the expression of cardiac genes particularly. Thus, it can be speculated that TEAD1 was a necessary part in the formation of myocardium. Furthermore, it was observed that only the growth of cardiac muscles, and not differentiation, was affected. Therefore, TEAD1 is involved in maturation of the embryonic heart [14]. Nevertheless, there are controversial conclusions about the functions of TEAD2. In 2007, Kaneko’s team reported that inactivation of the TEAD2 gene remarkably enhanced the risk of a certain defect in neural tube closure called exencephaly in mice [21]. However, one year later, the TEAD2 null embryos were reported to be normal by Sawada *et al.* [22]. Furthermore, in the subsequent study, nearly half of the embryos with TEAD1^+/−^/TEAD2^−/−^ showed exencephaly, the defects which appeared in Kaneko’s study. The reasons for these contradictions remained unknown. Accordingly, the latter research supported the overlap roles of TEAD1 and TEAD2. That the TEAD1^−/−^/TEAD2^−/−^ double mutant mice showed much more severe and widespread defects also suggested the functional redundancy of TEAD1 and TEAD2 [22].

Functional studies of TEAD3 in mice have not been reported, except for some evidence supporting that it played a role in DNA methylation [23]. TEAD4 is involved in the formation of the trophectoderm, a precursor of placenta. Therefore, the embryos failed to implant into the uterine endometrium with TEAD4 silencing [18,24]. However, the disruption of TEAD4 did not affect the normal development of embryos after implantation. These findings suggested that TEAD4 was particularly required for the specification of trophectoderm.

## 4. The Oncogenic Function of TEAD Proteins in Malignancies

TEAD transcription factors are not only crucial for the development process, but also play promoting roles in several cancer types. Multiple genes strongly correlated with tumorigenesis were regulated by TEADs, including secretory proteins connective tissue growth factor (CTGF) and Cyr61 [6], AXL receptor tyrosine kinase [25], Myc and survivin [26,27]. TEADs were thus proposed to be key mediators of normal growth and tumorigenesis [28]. Accordingly, it was shown that TEADs were over-expressed in breast cancer stem cells [29], breast cancers [30,31], fallopian tube carcinoma [32], germ cell tumors [33], renal cell carcinoma [34], medulloblastoma [35] and gastric cancer (GC) [36]. In addition, TEADs can not only elicit the hyper-activation of the mesothelin gene, but also serve as well-known tumor biomarkers [37]. Reports in this area are mostly focused on TEAD1 and TEAD4. TEAD1 expression with 300 times over the baseline was observed in Kaposi sarcoma [38]. In addition, when a prostate cancer patient is detected with a high TEAD1 expression, the clinical outcome of this patient might be poor [39]. TEAD1 was involved in the regulation of prostate epithelial cell differentiation and epithelial morphogenesis, such as the regulation of cell adhesion to the basement membrane. In both PC3 cells and patients’ samples, TEAD1 showed a high expression level, which was correlated with poor prognosis in prostate cancer patients [39]. The expression and clinical correlation of TEADs are summarized in Table 1.

**Table 1:** Summary of TEAD family expression in malignancies. In nearly all the cancer types, the expression of TEADs are up-regulated, suggesting a tumor-promoting role of TEADs. In gastric, colorectal, breast and prostate cancer, the upregulation of TEADs correlates with poor survival in patients.

Cancer Type	TEAD Family	Expression in Cancers	Prognostic Marker	Reference
Gastric cancer	TEAD1/4	up-regulated	√	[40,41]
Liver cancer	YAP-TEAD	up-regulated	–	[42,43,44]
Colorectal cancer	TEAD4	up-regulated	√	[45]
Lung cancer	YAP-TEAD	up-regulated	–	[46]
Breast cancer	TEAD4	up-regulated	√	[31]
Fallopian tube carcinoma	TEAD4	up-regulated	–	[32]
Ovarian cancer	TEADs	up-regulated	–	[47]
Germ cell tumor	TEAD4	up-regulated	–	[33]
Prostate cancer	TEAD1	up-regulated	√	[39]
Renal cell carcinoma	TEAD1	up-regulated	–	[34]
Medulloblastoma	TEAD1	up-regulated	–	[35]
Cutaneous melanoma	TEAD1/4	up-regulated	–	[30]
Kaposi carcinoma	TEAD1	up-regulated	–	[38]

### 4.1. YAP and TAZ Are General Transcription Coactivators for TEAD Transcription Factors

YAP and TAZ are components in the Hippo signaling pathway. The Hippo pathway was first discovered in a fruit fly (*Drosophila melanogaster*) that controlled the cell growth and organ size in diverse species, and its knockout caused tumorigenesis [48]. The name Hippo was from an experiment where the silencing of the members in this pathway caused an overgrowth of flies which resemble the skin of a Hippopotamus [49]. The main function of the Hippo pathway is to inhibit proliferation and to promote apoptosis, thereby to limit the overgrowth of organs [50]. It was identified to be conserved in mammals and involved in tumorigenesis of different kinds of cancer types [51,52]. Thirteen proteins were included in the Hippo pathway as kernel members (MST1/2, SAV1, LATS1/2, MOB1A, MOB1B, YAP1, TAZ, and TEAD1–4) [51]. SAV1 provides the supporting stage for MST1/2 and LATS1/2 to aggregate and promotes phosphorylation of LATS1/2 by MST1/2. MOB raises the activity of LATS1/2 and facilitates its interaction with LAST1/2. Then, the activated LAST1/2 phosphorylates the downstream transcriptional coactivators, YAP and TAZ, and quenches their function. The phosphorylation of YAP/TAZ suppresses their activity by creating the 14-3-3 binding site, which secludes YAP/TAZ in the cytoplasm [26,53].

YAP and TAZ have been identified as terminal effectors of the Hippo pathway [54], because they are the most pivotal step for the core kinase cassette of the pathway. YAP and TAZ shuttle from the cytoplasm to the nucleus, where they induce the expression of proliferation-promoting and anti-apoptotic genes through interaction with TEADs [55]. About 75% of purified TEAD2 was reported to be associated with YAP in mammals [56]. TEADs were also identified to be the most potently activated targets of YAP1 from the human transcription factor library [6]. TEADs are considered as key transcription factor partners for YAP/TAZ in regulation of gene expression [6,56,57]. Knockdown of TEADs or disruption of the interaction of YAP-TEAD diminished YAP-dependent gene transcription and oncogenic transformation both *in vitro* and *in vivo* [58,59,60]. The dysfunction of the Hippo pathway, which increased YAP/TAZ activity, induced oncogenic transformation due to the activation of transcription factors including TEAD family members.

Sonic hedgehog (Shh)-dependent medulloblastoma is a kind of tumor arising from cerebellar granule neuron precursors (CGNPs). In this kind of tumor, YAP and TEAD1 were over-expressed [35]. The Shh pathway stabilizes both TEAD1 and IRS1. This pathway can also lead to YAP1 overexpression, stabilization, and nuclear translocation. YAP1, TEAD1 and IRS1 interact with each other to co-regulate downstream gene expression [35].

In HEK293T cells, TEAD family transcription factors were identified as the major TAZ interacting transcription factors. TEADs were confirmed to be indispensable for TAZ to stimulate cell proliferation, migration and epithelial-to-mesenchymal transition (EMT). Impressively, *CTGF*, a gene regulating cell adhesion, proliferation and migration, was revealed as a direct target for TAZ and TEADs [61].

In breast cancer and melanoma, YAP interacts with TEADs to promote multiple processes such as proliferation, transformation, migration and invasion, which are important for tumor progression and metastasis. It was thus indicated that increased YAP and TEADs activities played a crucial role in cancer development and metastasis. Intriguingly, the increased transcriptional activity of TEADs was significantly correlated with the metastatic potential of breast cancer and melanoma [30]. In breast cancer, a novel mechanism of TEADs to regulate nuclear retention and transforming ability of TAZ has been revealed. Mechanistically, TAZ mutants defective in interaction with TEADs failed to accumulate in the nucleus. Knockdown of TEADs quenched TAZ-mediated oncogenic transformation of MCF10A cells [62]. More interestingly, TEAD4 alone was able to transform MCF10A cells with efficiency comparable with that of TAZ, which raised the possibility that TEAD4 itself may act as an oncogene during cancer development [62]. Recently, TEAD4 has been revealed to be a potential therapeutic target and prognostic biomarker in breast cancer. Its overexpression promoted tumor growth, but stable TEAD4 knockdown dramatically suppressed viability both *in vitro* and *in vivo*. Furthermore, TEAD4 was identified in collaboration with KLF to promote cell proliferation and tumor growth in triple negative breast cancer (TNBC) partly by inhibiting *p27* transcription [31]. In breast cancer cells, Lai *et al.* defined the TAZ/TEAD-Cyr61/CTGF signaling pathway as an important modifier for the Taxol response. They identified that the interaction of TAZ with TEAD transcription factors was essential for TAZ to activate the *cyr61*/*CTGF* promoters and induce Taxol resistance [63].

In high-grade clear cell renal cell carcinoma (RCC), the expression of SAV1, an upstream factor in Hippo pathway, was down-regulated. SAV1 suppressed the transcriptional ability of YAP1 and TEAD3 complex in RCC [64]. Recently, in clear cell RCC, TEAD1 was reported to be highly expressed. With ChIP-qPCR on screened regions containing TEAD-binding motifs within the promoter region, YAP and TEADs were simultaneously present on the promoter regions of *Myc*, *EDN1*, as well as *EDN2* genes [34].

In ovarian cancer initiated cells (OCICs), TEAD1, TEAD3 and TEAD4 were found to be expressed at significantly higher levels than in differentiated ovarian cancer cells. TEADs and YAP were further shown to be required for maintaining the expression of specific genes that were involved in stemness and chemoresistance of OCICs [47]. Most recently, miR-129-5p was revealed to function as a tumor suppressor miRNA that directly inhibited YAP/TAZ expression. This would result in the inactivated TEADs and subsequently exert suppression effects on cell proliferation, survival and tumorigenicity in ovarian cancer. In ovarian cancer cells, ectopic expression of miR-129-5p significantly inhibited TEAD-dependent luciferase activity. In addition, either depletion of YAP or TAZ in ovarian cancer suppressed the TEAD transcriptional activity, which was induced by antagomiR-129-5p [65].

In hepatocellular carcinoma (HCC), Liu *et al.* demonstrated a dominant-negative TEAD molecule that potently suppressed YAP1-induced hepatomegaly and tumorigenesis. They also confirmed that Verteporfin, a small molecule, could inhibit YAP-TEAD interaction and suppress YAP-induced overgrowth. Subsequently, Bai *et al.* suggested that YAP increased chemosensitivity of HCC cells by modulation of p53. Furthermore, they confirmed that this occurrence is only because of the TEAD binding domain [42]. Recently, a new regulatory mechanism of YAP1/TEAD2 by LATS2-mediated phosphorylation was identified in HCC. YAP was found to bind directly to TEAD2 and LATS2 inhibition-mediated dephosphorylation increased the YAP1/TEAD2 association. This resulted in YAP1/TEAD2 transcriptional activation, which in turn promoted cell invasion in HCC cells [43]. Another regulatory mechanism of YAP2/TEAD4, which was involved in HCC tumorigenesis and drug resistance, was revealed by Mao *et al.* They found that SIRT1 deacetylated YAP2 protein in HCC cells, which strengthened the YAP2/TEAD4 association and enhanced YAP2/TEAD4 transcriptional activation, and thus finally promoted cell growth in HCC [44].

In two independent colon cancer databases, TAZ-TEAD complexes inducing the upregulation of AXL and CTGF have been identified. AXL and CTGF were found, when combined with TAZ mRNA expression, to form a better prognostication in these two independent colon cancer patient databases [66]. Interestingly, in colorectal cancer (CRC), TEAD4 nuclear expression was suggested as a biomarker for CRC progression and poor prognosis. Both *in vitro* and *in vivo* study using CRC cells, the epithelial-mesenchymal transition followed with cell mobility change were decreased after TEAD4 silencing. Furthermore, with rescued expression of both wide type TEAD4 and a Y429H mutant (a mutation that impairs the interaction between TEAD4 and YAP/TAZ), they pointed out a YAP-independent manner of TEAD4 function in CRC, thus providing a novel mechanism of TEAD4 transcriptional regulation and its oncogenic role in CRC, independent of Hippo pathway [45].

### 4.2. p160 Family and TEAD Transcription Factors in Malignancies

Among the initial factors that interacted with nuclear receptors (NRs) in a highly ligand-dependent manner [67], three proteins with approximately 160 kDa were SRC1, TIF2, and RAC3 [68]. Although encoded by separate genes, they were highly homologous and capable of enhancing receptor-dependent transcriptional activation [68]. These three proteins constituted p160 family of coactivators or steroid receptor coactivator (SRC). SRC1, initially identified as a nuclear receptor coactivator, was found to interact with TEAD4. The interaction was mediated by the basic helix-loop-helix/Per-Arnt-Sim (bHLH-PAS) domain located in the N-terminus of SRC1 which was highly conserved. Moreover, all three members, SRC1, TIF2, and RAC3, have the ability to activate the transcription which requires the presence of the bHLH-PAS domain [69,70]. In fact, this domain is not only physically essential for binding, but also a requirement for enhancing the transcriptional activation of the interaction between p160 family proteins and TEADs [69]. And when interacting with TEADs, the p160 family of nuclear coactivators may also participate in normal development [69].

### 4.3. vgll Proteins and TEAD Proteins in Cancers

Vestigial-like (vgll) proteins are one group of TEAD transcriptional coactivators. The name derived from Vestigial (Vg), the transcriptional coactivator in *Drosophila*, and Vg is a main regulator in wing development [71,72]. Vg does not possess a DNA-binding domain and it pairs with the transcription factor Scalloped (Sd) to regulate gene expression. There is a short motif of about 26 animo acids in Vg and this motif is necessary and sufficient to interact with Sd. This motif is conserved in four mammalian proteins. It interacts with TEAD transcription factors to activate the downstream gene expression [2]. There are four members in vgll family in vertebrates named vgll 1–4.

Among the members, vgll1, also known as Tondu (TDU), was the first identified in human fetal kidney and lung tissues [72]. Despite containing a varied primary sequence, vgll1 interacts with TEADs in a manner similar to the oncogene YAP/TAZ. Functionally, the vgll1-TEADs complex up-regulated the expression of IGFBP-5, a proliferation-promoting gene, and facilitated anchorage-independent cell proliferation [2,73]. vgll2, also named VITO-1, is specifically expressed in skeletal muscle. Like vgll1, vgll2 interacts with TEAD1. Functionally, vgll2 may optimize the coactivator recruitment to TEAD1 during muscle differentiation [74]. vgll3, also called VITO-2, was detected in placenta and human fetal pancreas [75]. With a yeast two-hybrid assay, the murine homolog vgll3 interaction with TEAD1 has been verified [76]. vgll3 also potentially plays a role in cancers; it supported the progression of soft-tissue sarcoma and also the development of myxoinflammatory fibroblastic sarcoma, and possibly played a tumor suppressor role in ovarian cancers [77,78,79]; however, whether TEADs were involved is still not clear.

vgll1-3 contains the entire Sd/TEAD-interacting motif, whereas vgll4 has two partially conserved TEAD-interacting motifs [73]. vgll4 is believed to be a putative transcriptional co-regulator involved in cell survival pathway. It acts in the nucleus and modulates cell signals to prevent apoptosis, or it may be exported out of the nucleus to carry out a transcription-independent function mediating cell survival. The overexpression of vgll4 leads to a significantly higher proportion of live cells [80]. The features of cell survival promoting were also illustrated by evidence that vgll4 played a role in regulating cell apoptosis [81]. However, it is still unclear whether TEADs were involved in these processes. Due to the structural similarity, vgll4 was also reported to be a tumor suppressor by binding TEADs and competing with YAP/TAZ in GC [40], and this principle was also used in a new anticancer therapy [82]. Furthermore, an aberrant level of vgll4 mRNA inversely correlated with gastric tumor size, tumor stage, lymphatic invasion and lymph node metastasis [82]. A strong correlation between downregulation of vgll4 and poor outcomes of pancreatic cancer patients also suggested a tumor repressor role of vgll4 [83].

In other malignancies, vgll4 was still considered as a suppressor for YAP activity via competition for TEAD binding. Koontz *et al.* suggested vgll4 can be exploited as a potent inhibitor for YAP oncoprotein in mammals [84]. In lung cancer, vgll4 was validated to be a novel tumor suppressor through negatively regulation the association of YAP-TEAD complex. The data demonstrated that vgll4 and TEAD4 co-localized in the nucleus and had strong binding affinity with each other. Furthermore, vgll4 abolished the transcriptional activity of TEADs by competing with YAP and exerted its growth-inhibition function through two TDU domains [46]. In GC, a peptide mimicking vgll4 function was used in therapy as a YAP antagonist, and vgll4 was found to directly compete with YAP for binding to TEADs in GC; the peptide mimicking vgll4 function potently inhibited tumor growth *in vitro* and *in vivo* [82].

In GC, increased TEAD1 [40] and TEAD4 [41] have been proposed as prognostic biomarkers in GC patients. Accordingly, TEAD1 was involved in a feedback mechanism of miR-222/vgll4/TEAD1 loop, in which miR-222 decreased the activity of vgll4. vgll4 is a transcriptional inhibitor that represses the YAP-TEAD complex by competing with YAP directly for binding TEADs. Furthermore, TEAD1 was found to bind the miR-222 promoter physically, and induced the expression of miR-222. Thus high expression of TEAD1 indicated poor outcomes of GC patients [40]. On the other hand, DNA hypo-methylation at the TEAD4 promoter was discovered, which resulted in the activation of TEAD4 expression. As a consequence, a high level of TEAD4 suggested a poor survival in GC patients [41].

Shen *et al.* found that miR-130a, a direct downstream target of YAP, effectively suppressed the tumor suppressor function of vgll4. Thus, the YAP-miR-130a-vgll4 positive feedback loop was proposed to function downstream of Hippo signaling to mediate potent responses, and play an important role in liver tumorigenesis [85]. Moreover, TEAD1 physically binds to the promoter region of miR-222 and promotes the transcription of this miRNA, which further downregulates the expression of vgll4 protein. Thus, YAP-induced cell proliferation and tumorigenesis is induced through vgll4 downregulation, [40].

## 5. The Downstream Effectors of TEAD Transcription Factors

**Figure 1:**
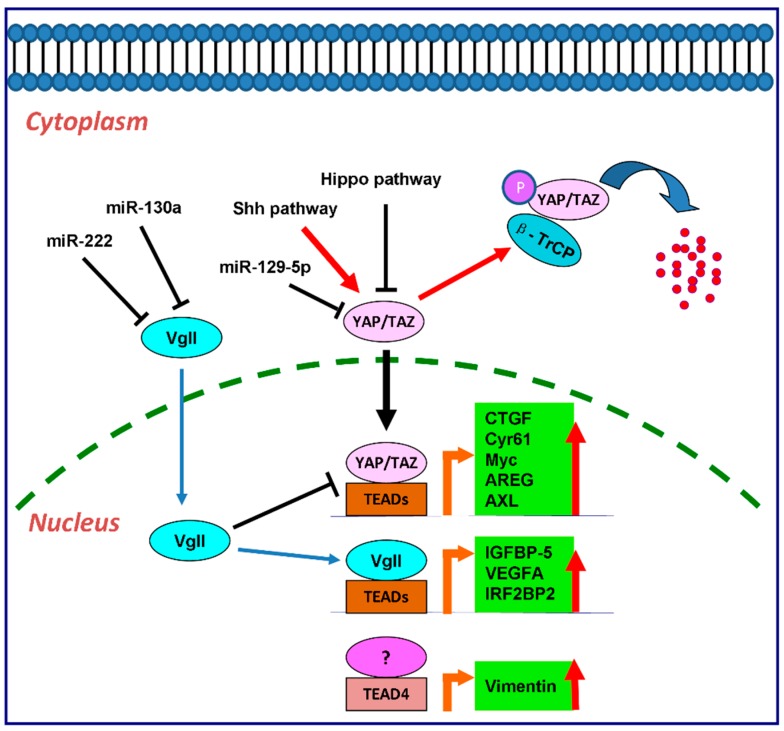
The regulatory cascade of TEAD family in cancer cells as transcription factors. YAP/TAZ, vgll, and p160 family proteins are the main binding partners for TEADs to activate the downstream transcription as transcription co-activators. *CTGF*, *Cyr61*, *Myc*, *AREG* and *AXL* are the downstream targets of YAP and TAZ by interaction with TEADs. vgll directly regulates the expression of *IGFBP-5*, *VEGFA* and *IRF2BP2* through TEADs. In colon cancer, TEAD4 also regulates Vimentin expression in a YAP-independent manner.

The downstream effectors of TEAD transcription factors included *Cyr61* [63,86,87], *CTGF* [61], *Myc* [26,88], *AREG* [89], *Gli2* [87], *Vimentin* [45], *AXL* [25], as shown in Figure 1.

### 5.1. CTGF and Cyr61

Both Cyr61 (CCN1) and CTGF (CCN2) belong to the CCN (Cyr61, CTGF, NOV) family, which include a type of cysteine-rich extracellular matrix protein. Both of them function as ligands of the integrins to regulate a great number of cellular activities, not only in cell proliferation, differentiation and apoptosis [90,91], but also in inflammation and tumorigenesis. TEADs directly targeted *CTGF* and *Cyr61*. The promoter region of *CTGF* gene contains several GGAATG motifs for TEAD-binding while two putative TEAD-response elements in the *Cyr61* promoter region have been identified. Functionally, knockdown of CTGF partially impaired the oncogenic property of YAP-TEAD complex [6]. Knockdown of Cyr61 and CTGF reversed the drug-resistant phenotypes caused by TAZ [63]. Moreover, TEADs are required for TAZ to activate the *CTGF* promoter, and thereby to accomplish the TAZ-induced cell proliferation process [61].

### 5.2. Myc

Myc or c-Myc, which is known as a functionally conserved transcriptional regulator, controls various aspects of cell biology by activating a great number of target genes [92,93]. In mammals, *Myc* functions as an oncogene in numerous types of tumors, and is also a necessary factor for growth during development and in regeneration. Nevertheless, Myc is not only normally associated with biological activities, such as hepatocyte proliferation [26], but also cooperates with YAP and acts as an oncogene to stimulate tumor growth in nude mice [94]. With ChIP-seq to analysis the YAP/TAZ-binding sites in breast cancer, Zanconato *et al.* found that Myc was one of the targets that potentially is able to amplify the effects of YAP/TAZ. They suggested Myc exhibited an important functional effector of YAP/TAZ. However, Myc alone could not fully recapitulate the biological effectiveness of YAP/TAZ [95]. Recently, Hiroshi *et al.* established an *in vitro* model system and demonstrated the cell competition phenomenon in mouse NIH3T3 embryo fibroblast cells. They suggested that TEADs directly up-regulated Myc expression and cells with increased Myc expression became super-competitors [96].

### 5.3. AREG

Amphiregulin (AREG), a member of the epidermal growth factor (EGF) family, was identified in 1988 by Shoyab *et al.* AREG was defined for the first time as a bi-functional growth factor for its capacity of inhibiting proliferation in certain tumor cell lines while promoting cell viability of normal cells such as fibroblasts and keratinocytes [97]. AREG is known as a proliferation-stimulating factor in most cells. This effect is majorly mediated via its binding and activation of a wild-type expressed transmembrane tyrosine kinase called epidermal growth factor receptor (EGFR) [98]. *AREG* was also identified as a target gene of YAP [89]. The induction of AREG by YAP could be detected only under the condition of EGF starvation. However there is no TEAD-binding element in the AREG promoter. Thus, the transcription factor mediation of AREG induction remains vague. Seemingly, the main function of the Hippo pathway and YAP is cell-autonomous *in vivo*. Thus, AREG has been implicated as an effector of YAP in conferring growth factor-independent growth, however whether TEADs are involved in the AREG transcription is unclear [89].

### 5.4. AXL

AXL is another direct target of the YAP-TEAD complex [25]. There are four putative TEAD-binding sites located within 1200 bp upstream of the transcriptional start site of AXL. In HCC, AXL was confirmed to be a main mediator of YAP-dependent oncogenic activities [25].

### 5.5. Gli2

Gli2 belongs to transcriptional factor in Shh signaling pathway. Researchers have found that YAP1/TEAD1 can play a role in regulating the Gli2 expression. Gli2 can thereby play a role to activate downstream mediators in Shh-induced proliferation [35].

### 5.6. Vimentin

Vimentin, a 57 kDa intermediate filament protein, is normally expressed in mesenchymal-origin cells, such as myofibroblasts and endothelial cells [99]. In a physiological circumstance, Vimentin plays a fundamental role in cell adhesion, which makes it a key molecule in maintaining endothelial barrier integrity [100]. TEAD4 directly up-regulates Vimentin to alter the morphology and migration of CRC cells in a YAP-independent manner [45].

## 6. Conclusions and Future Directions

From the studies reviewed in this article, it is quite clear that TEAD transcription factors are strongly involved in human malignancies and play critical roles in cancer initiation and progression. The activation of TEADs as transcription factors requires the involvement of coactivators, such as YAP, vgll, and p160 family proteins to stimulate the transcription activity on the downstream target genes. It thus gives us a hint that disrupting the interaction between TEADs and their coactivators involved in tumorigenesis might be a promising strategy for future cancer therapies.

Hence, as TEADs are involved in cancer development, suppression of the interaction between TEAD family and their coactivators provide a promising choice for cancer intervention. Recently with the demonstration of the crystal structure of TEAD-YAP complex, a small molecule named Verteporfin was suggested to function as an inhibitor for the protein interaction [101]. Indeed, Verteporfin-induced disruption of the YAP-TEAD interaction has provided an optional choice for YAP-overexpression liver tumorigenesis [102]. Malignant pleural mesothelioma (MPM) is characterized by loss of function or mutations in the neurofibromin 2 (NF2) and the cyclin-dependent kinase inhibitor 2 genes. The Hippo pathway was activated by NF2, which resulted in the cytoplasmic retention of YAP and its co-activator TEAD. Furthermore, inhibition of the YAP-TEAD complex assembly has been suggested to stimulate MPM growth, offering clinical translational potential [103]. In YAP/TAZ-dependent breast cancers, combinations of dasatinib, fluvastatin, and pazopanib compounds with each other or with other anti-cancer drugs efficiently reduced the YAP/TAZ-TEAD-dependent reporter activity [104].

However, two important issues need to be addressed in the following studies. The first one is about the regulation of TEADs in tumorigenesis. With the deepening understanding on the oncogenic role of TEAD transcription factors, the activation of TEADs in promoting carcinogenesis is still elusive. DNA copy number aberrations, regulation from the transcription level, miRNA regulation from post-transcription level, or even post-translational modification might determine the expression and activation of TEADs. Secondly, with TEADs binding with different transcription coactivators to activate the expression downstream genes in a cell-context dependant manner, the full identification and investigation of the binding partners of TEADs will help us to elucidate the dual role of TEADs in physiological or pathological processes.
